# β2-microglobulin and cognitive decline: unraveling the mediating role of the Dunedin Pace of Aging methylation

**DOI:** 10.3389/fnagi.2025.1505185

**Published:** 2025-03-25

**Authors:** Yujun Ke, Ping Chen, Chunlan Wu, Qinqin Wang, Kai Zeng, Min Liang

**Affiliations:** ^1^Department of Anesthesiology, The First Affiliated Hospital, Fujian Medical University, Fuzhou, China; ^2^Department of Anesthesiology, National Regional Medical Center, Binhai Campus of the First Affiliated Hospital, Fujian Medical University, Fuzhou, China; ^3^Department of Oncology, Molecular Oncology Research Institute, The First Affiliated Hospital, Fujian Medical University, Fuzhou, China; ^4^Department of Oncology, National Regional Medical Center, Binhai Campus of the First Affiliated Hospital, Fujian Medical University, Fuzhou, China; ^5^Department of Rheumatology, The Affiliated Nanping First Hospital of Fujian Medical University, Nanping, China

**Keywords:** β2-microglobulin, DNA methylation, DunedinPoAm, cognitive function, mediation analysis

## Abstract

**Background:**

Progressive cognitive decline is inevitable with aging. Growing evidence links β2-microglobulin (B2M) to aging and cognitive decline. However, the current evidence is inadequate to establish a definitive association. This study aims to investigate the relationship between B2M levels and cognitive performance, together with the mediating effect of the pace of biological aging.

**Methods:**

Utilizing the 1999–2002 National Health and Nutrition Examination Survey (NHANES) database, cognitive performance was measured via the Digit Symbol Substitution Test (DSST), while the pace of biological aging was quantified using a new generation DNA methylation algorithm, Dunedin Pace of Aging methylation (DunedinPoAm). Weighted multivariable linear regression was used to explore the relationship between B2M levels and cognitive performance. Furthermore, subgroup analysis and interaction tests were performed to assess the relationship’s stability. Mediation analysis was conducted to investigate the mediating effect of DunedinPoAm on the association between B2M levels and cognitive performance.

**Results:**

The study included 1,267 participants aged 60 and over. After correcting for all confounders, for each one-unit increment in log-transformed B2M levels, the DSST score fell by 5.13 points (95%CI −9.03 to −1.24), while the level of DunedinPoAm increased by 0.04 (95%CI 0.01–0.07). The analysis of the trend test yielded identical results (*p* for trend <0.05). Additionally, across every subgroup analyzed, the correlation between B2M levels and cognitive performance was stable (*p* for interaction >0.05). Further mediation analysis showed that DunedinPoAm mediated 9.0% (95%CI 0.1–43.2%) of the association between B2M and cognitive performance.

**Conclusion:**

These findings suggested a substantial link between elevated B2M levels and cognitive decline among U.S. older adults, partly mediated through the faster pace of aging. This correlation highlights the potential of B2M as a biomarker for early detection and therapeutic intervention of aging-related cognitive decline.

## 1 Introduction

Aging is an unavoidable process characterized by the progressive decline of all bodily functions. These degenerative changes subsequently heighten vulnerability to age-related medical conditions, such as dementia (Kennedy et al., 2014). Aging stands as the main factor responsible for the onset of neurodegenerative disorders associated with dementia (Hou et al., 2019). Different individuals appear to age along different trajectories (Nie et al., 2022). Identifying an individual’s unique aging profile through biomarker analysis may reveal potential therapeutic targets to delay or reverse aging-related cognitive decline.

β2-microglobulin (B2M) is the soluble light chain component of the major histocompatibility complex class I (MHC-I) molecules. Clinicians have long noted anecdotal associations between elevated B2M levels and cognitive decline, with elevated B2M levels observed in patients with Alzheimer’s disease (AD) (Huang et al., 2023), dementia associated with human immunodeficiency virus (HIV) (Gates et al., 2016), and cognitive impairment following brain injury (Huo et al., 2022). Nevertheless, studies have demonstrated that when accounting for renal function, the correlation between B2M and cognitive function becomes statistically insignificant (Tin et al., 2023). Hence, the current evidence is inadequate to establish a definitive association between B2M and cognitive decline. Moreover, studies have indicated that B2M levels positively correlate with age (Dong et al., 2016; Yang et al., 2017). Cognitive decline, caused by elevated circulating levels of B2M, is also a prevalent indicator of aging. This prompts a significant inquiry: Is the elevation in B2M a causative factor or a resultant effect of aging?

The DunedinPoAm (Dunedin Pace of Aging methylation) is a new generation algorithm for quantifying biological aging based on DNA methylation. Through machine learning analysis of blood DNA methylation data, which is highly sensitive to aging-related variation, DunedinPoAm can precisely estimate the personalized pace of aging among individuals of the same chronological age (Belsky et al., 2020). Out of earlier epigenetic clocks, DunedinPoAm exhibits a more robust correlation with cognitive function and susceptibility to cognitive deterioration (Sugden et al., 2022).

Therefore, we first investigated the relationship between B2M levels and cognitive performance using clinical data from the National Health and Nutrition Examination Survey (NHANES), a large and statistically robust survey. Furthermore, we employed a mediation analysis model to investigate whether variations in B2M levels might indirectly influence cognitive performance by modulating the pace of biological aging, as measured by DunedinPoAm.

## 2 Methods

### 2.1 Study population

NHANES is a significant public health survey conducted by the National Center for Health Statistics (NCHS) that offers a vital data source for examining the health and nutritional condition of the U.S. population. NHANES employs a stratified multistage sampling methodology to obtain a representative sample of U.S. residents. Each participant provided written informed consent upon recruitment.

**Figure 1:**
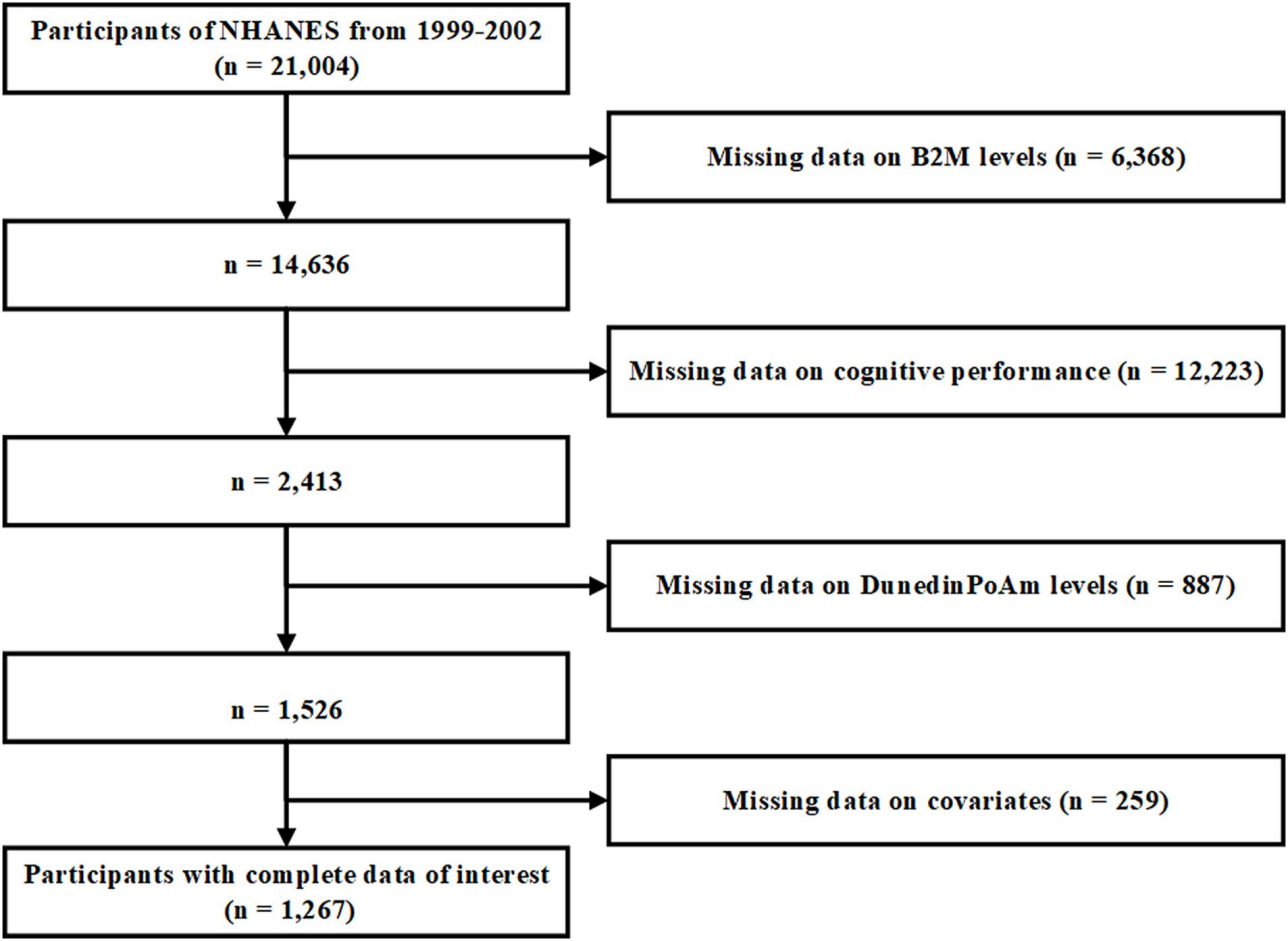
Flow chart of participants selection.

During the 1999–2002 survey period, NHANES assessed the cognitive performance of individuals aged 60 years or older. Additionally, DNA methylation and serum B2M levels were quantified. Our study participants were individuals with comprehensive and reliable data on cognitive test scores, serum B2M levels, and DunedinPoAm data. After excluding subjects with missing covariate information, our study cohort comprised 1,267 participants. The recruitment process is shown in Figure 1.

### 2.2 Measurement of B2M levels

B2M levels were the primary variable in the study. The measurements were conducted at the University of Maryland School of Medicine in Baltimore, Maryland, from 2018 to 2020. The B2M immunoassay was used to measure serum B2M on the Siemens Dimension Vista 1500, an automated multichannel analyzer manufactured by Siemens Healthcare Diagnostics. The upper and lower detection limits for B2M levels were 23.0 mg/L and 0.72 mg/L, respectively.

### 2.3 Assessment of cognitive performance

The cognitive performance of persons aged 60 and above was evaluated using the Digit Symbol Substitution Test (DSST), a reliable and widely accepted assessment of cognitive function (Joy et al., 2004). Participants were required to correctly encode a sequence of symbols within a time limit of 120 s. Higher scores were awarded for a more significant number of correct completions. The highest achievable score was 133 points, with higher levels suggesting better cognitive performance.

### 2.4 Measurement of DunedinPoAm

Samples from individuals aged 50 and above who had undergone whole-blood DNA purification were obtained for DNA methylation analysis. DNA methylation was analyzed at Duke University laboratory utilizing Illumina EPIC microbead chip arrays. Matrix generation of methylation raw data in IDAT format for subsequent preprocessing and normalization. DunedinPoAm data was analyzed using the specific code supplied in the DNA Methylation Array and Epigenetic Biomarkers Data Documentation.

### 2.5 Covariate information

This study included confounders that may affect the relationship between B2M and cognitive performance, categorized into four groups: sociodemographic characteristics, lifestyle factors, prevalent diseases in the elderly, and renal function (Tan et al., 2024). Sociodemographic characteristics included age (years); sex (male and female); ethnicity (non-Hispanic Black, non-Hispanic White, Mexican American, and other races); education level (less than high school, high school graduate, some college, and college graduate); body mass index (BMI; underweight: <18.5 kg/m^2^, normal weight: 18.5–24.9 kg/m^2^, overweight: 25.0–29.9 kg/m^2^, and obese: ≥30.0 kg/m^2^); and poverty income ratio (PIR; <1 and ≥1). Lifestyle factors encompassed smoking status (never: fewer than 100 cigarettes in a lifetime; former: more than 100 cigarettes in a lifetime and ceased smoking; current: more than 100 cigarettes in a lifetime and actively smoking) and alcohol consumption (no: fewer than 12 alcoholic beverages per year; yes: at least 12 alcoholic beverages per year). Prevalent diseases in the elderly encompassed hypertension and diabetes, both of which were characterized by a formal diagnosis from a physician. The latest KDIGO guidelines classified renal function based on the Urinary Albumin to Creatinine Ratio (UACR): normal (<30 mg/g), mild renal insufficiency (30–299 mg/g), and moderate to severe renal insufficiency (≥300 mg/g) (Levin et al., 2024).

### 2.6 Statistical analysis

The analyses were conducted using R (version 4.3.3). Considering the complex multistage probability sampling design of NHANES, referring to the NHANES Analytic Guidelines, the sample weights used in this study were the cardiac biomarker 4-year weights 1999–2002. Due to the skewed distribution of B2M levels, a natural logarithm (Ln) transformation was applied to treat it as a continuous variable for regression, subgroup, and mediation analyses. Alternatively, the data was categorized into quartiles (Q1, Q2, Q3, and Q4) to facilitate baseline and trend test analyses.

Participants were classified into four groups in baseline analysis according to the quartiles of serum B2M levels. Continuous variables were expressed as means ± standard deviations, whereas categorical variables were presented as percentages. Categorical and continuous variables were compared using Chi-squared and *t*-tests. Weighted multivariable linear regression was utilized to investigate the relationship between B2M levels and cognitive performance. The trend test employed integer values (1, 2, 3, and 4) across ascending exposure groups. There was no covariate adjustment for Model 1. Age, sex, and race were adjusted in Model 2. Model 3 was further adjusted for smoking status, alcohol consumption, educational level, BMI, PIR, hypertension, diabetes, and UACR. Subgroup analysis and interaction tests were performed to assess the stability of the relationship between B2M levels and cognitive performance across various subgroups while controlling for all confounding variables.

Mediation analysis was conducted using R’s “mediation” packages (version 4.3.3). Indirect effects, direct effects, and proportions of mediation were tested using thousands of bootstraps. Age, sex, race, smoking status, alcohol consumption, educational level, BMI, PIR, hypertension, diabetes, and UACR were all taken into account while adjusting the mediation analysis models.

## 3 Results

### 3.1 Baseline characteristics of participants

The cross-sectional study included 1,267 participants aged 60 and over. Table 1 summarizes the weighted baseline characteristics, categorized by quartiles of B2M levels. Significant differences were observed among quartiles regarding age, race, alcohol consumption, BMI, hypertension, diabetes, and UACR (*p* < 0.05). Compared to the cohort with lower B2M levels, the cohort with higher B2M levels demonstrated inferior cognitive performance and a faster aging pace, as assessed by the DunedinPoAm.

**Table 1:** Weighted baseline characteristics by quartiles of B2M levels.

Characteristics	Overall	Q1	Q2	Q3	Q4	*p* value
Age	70.32 ± 0.27	66.70 ± 5.71	69.01 ± 6.23	70.22 ± 7.12	74.92 ± 7.07	<0.001
Sex (%)						0.154
Male	43.38	45.12	48.50	42.19	37.70	
Female	56.62	54.88	51.50	57.81	62.30	
Race (%)						0.006
Mexican American	4.96	5.16	7.18	3.92	3.57	
Non-Hispanic Black	12.02	17.16	10.71	8.92	11.30	
Non-Hispanic White	71.16	61.63	67.70	77.80	77.49	
Other races	11.86	16.05	14.41	9.36	7.64	
Education level (%)						0.124
Less than high school	30.34	26.63	28.53	29.19	37.00	
High school graduate	28.03	27.39	25.00	28.61	31.12	
Some college	21.78	23.83	20.50	23.43	19.36	
College graduate	19.85	22.15	25.97	18.77	12.52	
Smoking status (%)						0.921
Never	46.83	44.99	46.70	47.31	48.33	
Former	41.02	43.37	40.00	39.55	41.16	
Current	12.15	11.64	13.30	13.14	10.51	
Alcohol consumption (%)						<0.001
No	39.32	30.97	33.49	39.88	52.95	
Yes	60.68	69.03	66.51	60.12	47.05	
BMI (%)						0.029
Normal	28.47	33.90	25.14	29.22	25.62	
Obese	32.73	31.94	32.50	29.52	36.96	
Overweight	37.66	33.20	41.93	41.15	34.36	
Underweight	1.14	0.96	0.43	0.11	3.06	
PIR (%)						0.064
<1	13.82	8.77	14.30	12.57	19.65	
≥1	86.18	91.23	85.70	87.43	80.35	
Hypertension (%)	51.61	49.87	40.47	50.68	65.44	<0.001
Diabetes (%)	14.27	13.04	9.41	13.45	21.19	0.003
UACR						<0.001
<30	81.33	88.25	85.05	84.89	67.11	
30–300	15.96	11.04	13.90	12.97	25.95	
≥300	2.71	0.71	1.05	2.14	6.94	
DSST score	45.44 ± 0.71	49.75 ± 18.18	47.72 ± 18.42	46.83 ± 17.06	37.96 ± 15.72	<0.001
DunedinPoAm	1.10 ± 0.01	1.09 ± 0.10	1.09 ± 0.08	1.10 ± 0.09	1.13 ± 0.10	0.001

Continuous variables were presented as mean ± SE. Categorical variables were presented as (%). BMI, body mass index; PIR, the ratio of family income to poverty; UACR, Urinary Albumin to Creatinine Ratio; DSST, Digit Symbol Substitution Test; DunedinPoAm, Dunedin Pace of Aging methylation.

### 3.2 Association between B2M levels and cognitive performance

**Figure 2:**
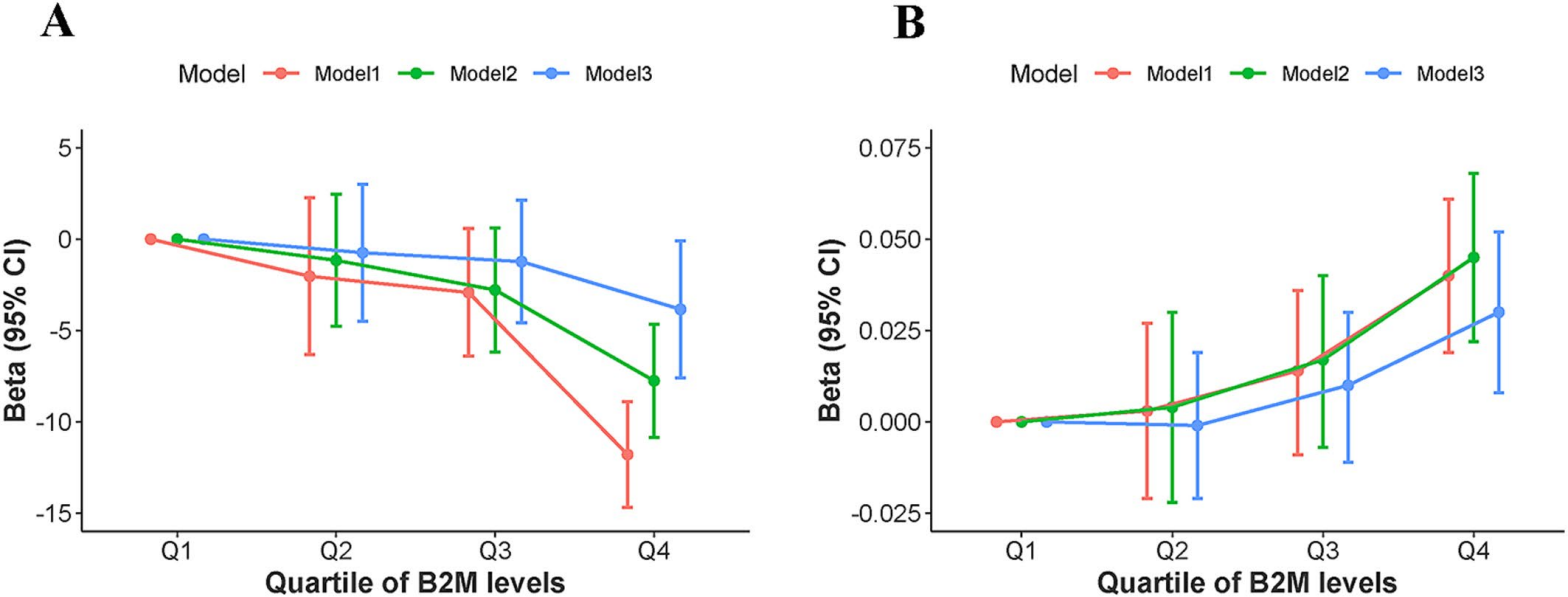
Trend test analysis of the association between B2M level quartiles and **(A)** Cognitive performance, **(B)** DunedinPoAm. Model 1: unadjusted model; Model 2: adjusted for age, sex, and race; Model 3: adjusted for age, sex, race, smoking status, alcohol consumption, educational level, body mass index, the ratio of family income to poverty, hypertension, diabetes, and Urinary Albumin to Creatinine Ratio. CI, confidence interval; Q, quartile.

The relationship between B2M levels and cognitive performance among U.S. older adults was demonstrated in Table 2 and Figure 2. Table 2 illustrated that for each one-unit increment in log-transformed B2M levels, the DSST score fell by 13.95 points in Model 1 (*β* = −13.95, 95%CI −17.55 to −10.34, *p* < 0.001) and by 9.49 points in Model 2 (*β* = −9.49, 95%CI −12.77 to −6.20, *p* < 0.001). After correcting for all confounders, Model 3 showed that the negative connection between B2M levels and cognitive performance was attenuated but remains statistically significant; for each one-unit increment in log-transformed B2M levels, the DSST score fell by 5.13 points (*β* = −5.13, 95%CI −9.03 to −1.24, *p* = 0.015). The analysis of the trend test yielded identical results. All three models exhibited a worsening trend in cognitive performance correlated with elevated levels of B2M (*p* for trend <0.05) (Figure 2).

**Table 2:** Association of B2M levels with cognitive performance and DunedinPoAm.

	Model 1		Model 2		Model 3	
	*β* (95% CI)	*p* value	*β* (95% CI)	*p* value	*β* (95% CI)	*p* value
DSST score	−13.95 (−17.55, −10.34)	<0.001	−9.49 (−12.77, −6.20)	<0.001	−5.13 (−9.03, −1.24)	0.015
DunedinPoAm	0.05 (0.02, 0.08)	<0.001	0.05 (0.03, 0.08)	<0.001	0.04 (0.01, 0.07)	0.015

Model 1: unadjusted model; Model 2: adjusted for age, sex, and race; Model 3: adjusted for age, sex, race, smoking status, alcohol consumption, educational level, body mass index, the ratio of family income to poverty, hypertension, diabetes, and Urinary Albumin to Creatinine Ratio. CI, confidence interval; DSST, Digit Symbol Substitution Test; DunedinPoAm, Dunedin Pace of Aging methylation.

### 3.3 Subgroup analysis

**Figure 3:**
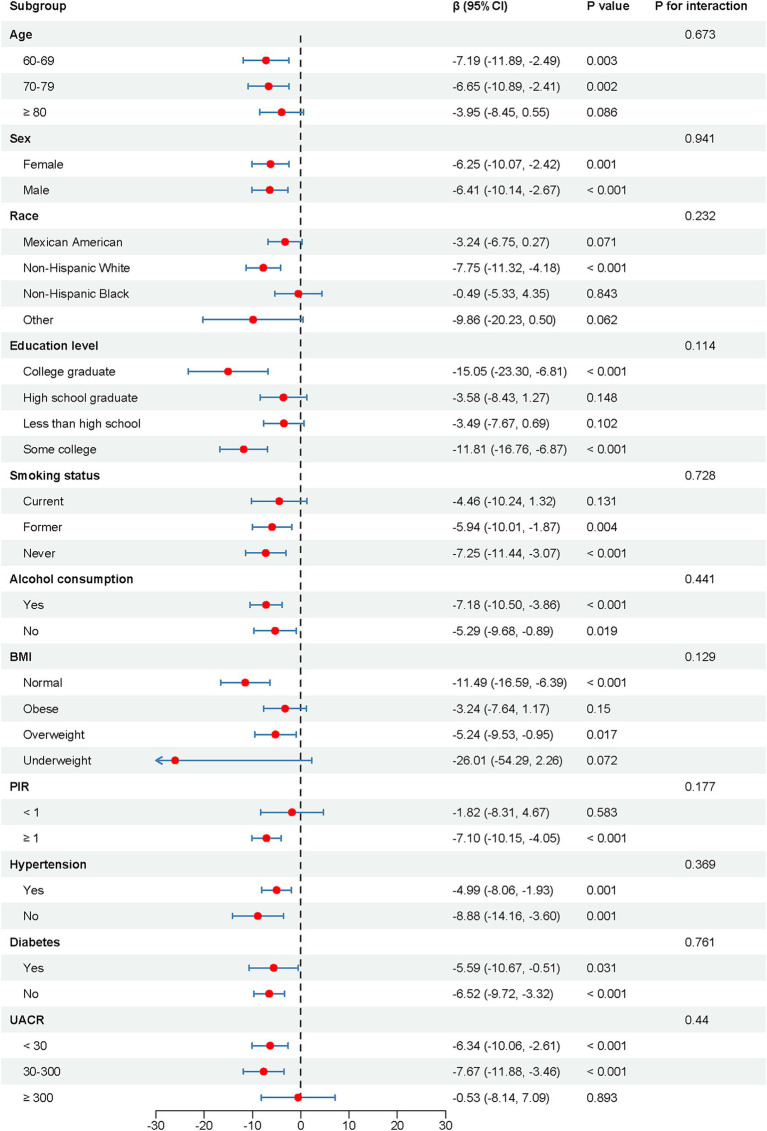
Subgroup analysis and interaction tests of the association between log-transformed B2M levels and cognitive performance. CI, confidence interval; BMI, body mass index; PIR, the ratio of family income to poverty; UACR, Urinary Albumin to Creatinine Ratio.

Figure 3 illustrates the results of the subgroup analysis and interaction testing. Across every subgroup analyzed, the correlation between B2M levels and cognitive performance was stable, with no significant interaction effects detected (*p* for interaction >0.05). Among the subgroups aged 60–69 and 70–79, Non-Hispanic White individuals, with educational attainment of college graduate and some college, comprising former and never smokers, possessing normal and overweight BMI, with a PIR exceeding 100%, and UACR values of <30 and 30–300, as well as across all subgroups defined by gender, alcohol consumption, hypertension, and diabetes, a trend indicated worsening cognitive performance correlated with elevated B2M levels (*p* < 0.05).

### 3.4 Association between B2M levels and DunedinPoAm

As shown in Table 2, for each one-unit increment in log-transformed B2M levels, the level of DunedinPoAm increased by 0.05 in Model 1 (*β* = 0.05, 95%CI 0.02–0.08, *p* < 0.001), 0.05 in Model 2 (*β* = 0.05, 95%CI 0.03–0.08, *p* < 0.001), and 0.04 in Model 3 (*β* = 0.04, 95%CI 0.01–0.07, *p =* 0.015). The trend test consistently indicated that the pace of aging accelerates with rising B2M levels (*p* for trend <0.05) (Figure 2).

### 3.5 Mediating analysis

**Figure 4:**
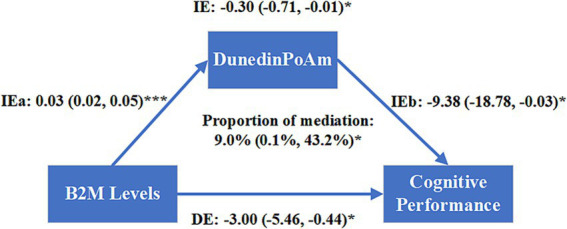
Mediation analysis of the association between log-transformed B2M levels and cognitive performance mediated by DunedinPoAm. Models were adjusted for age, sex, race, smoking status, alcohol consumption, educational level, body mass index, the ratio of family income to poverty, hypertension, diabetes, and Urinary Albumin to Creatinine Ratio. The effect of each path was presented as standardized regression coefficient (95% confidence interval). IE: the indirect effect; DE: the direct effect; IE = IEa × IEb; the total effect = IE + DE; Proportion of mediation = (indirect effect/total effect) × 100% = (IE/(IE + DE)) × 100%. *p < 0.05, **p < 0.01, ***p < 0.001.

As shown in Figure 4, the direct effect of B2M on cognitive performance was −3.00 (95%CI −5.46 to −0.44, *p* = 0.020). The indirect effect of B2M on DunedinPoAm was 0.03 (95%CI 0.02–0.05, *p* < 0.001) and the indirect effect of DunedinPoAm on cognitive performance was −9.38 (95%CI −18.78 to −0.03, *p =* 0.042). The indirect effect of B2M on cognitive performance mediated by elevated DunedinPoAm levels was −0.30 (95%CI −0.71 to −0.01, *p =* 0.036). DunedinPoAm mediated 9.0% (95%CI 0.1–43.2%, *p* = 0.044) of the association between B2M and cognitive performance.

## 4 Discussion

In this study, we have presented two primary and novel discoveries. We established a clear correlation between elevated B2M levels and declined cognitive performance among U.S. older adults. Furthermore, the pace of aging, as measured by DunedinPoAm, plays a mediating role in the relationship between B2M levels and cognitive performance.

Prior clinical research, which had limited sample numbers, has indicated that increased levels of B2M are linked to cognitive decline (Yang et al., 2017; Załęska-Kocięcka et al., 2017). However, specific research indicates that the correlation between B2M and cognitive function is contingent upon renal function (Tin et al., 2023). Our study, employing a substantial clinical sample from the NHANES database spanning 1999–2002 and controlling for factors that may influence cognitive function, especially renal function, revealed a significant association between higher B2M levels and a deterioration in cognitive capacity. Additionally, we performed subgroup analysis and interaction tests, revealing that this link remains consistent across all analyzed groupings. These findings present convincing clinical evidence. B2M can cross the blood–brain barrier (BBB) and cause harm to the central nervous system through different mechanisms independent of the MHC-I molecule. B2M, an endogenous NMDAR antagonist, impairs synaptic function by preventing glutamate from binding to its postsynaptic membrane receptors (Gao et al., 2023). Additionally, elevated B2M increases amyloid pathology and neurodegeneration in AD (Zhao et al., 2023). Furthermore, B2M affects nerve cell development and hippocampus neural progenitor cell self-renewal (Smith et al., 2015). Moreover, B2M participates in cognitive impairment by triggering neuroinflammation linked to the NLRP3 inflammasome (Chen et al., 2023). Collectively, the evidence presented substantiates our conclusion that increased B2M levels constitute an independent risk factor for cognitive deterioration.

DunedinPoAm, functioning as an epigenetic clock that quantifies the pace of biological aging, is fundamentally different from other clocks, such as PhenoAge (Levine et al., 2018) and GrimAge (Lu et al., 2022), which gauge the total extent of biological aging from birth to a specific moment. DunedinPoAm, conversely, emphasizes estimating the rate of biological aging at a specific moment and has heightened sensitivity to responses from senescence-related interventions, rendering it particularly adept at evaluating short-term changes in senescence and the consequences of therapies (Moqri et al., 2023). Our findings showed that DunedinPoAm consistently increased alongside rising B2M levels, suggesting that B2M may, to some extent, accelerate the pace of aging. A landmark animal experiment utilizing heterochronic parabiosis showed that B2M in aged animals’ blood drives the development of aging phenotypes in young mice’s brains (Smith et al., 2015). This study identified B2M as a pro-aging factor consistent with our findings. Indeed, GWAS has established a connection between the MHC locus on chromosome 6p21 and degenerative disorders associated with aging (Jeck et al., 2012). Mechanistically, the aging process depends on the combination of mechanisms within and between cells; B2M can modify cell communication and transmit aging signals between different cell types (Ma et al., 2022). In addition, B2M may lead to cellular senescence by activating the TLR4/MyD88/NF-κB signaling pathway in hippocampus neurons (Zhong et al., 2020). Overall, B2M, as a significant determinant in the aging process, is crucial in regulating the biological aging rate.

Given these findings, we proceeded to carry out a mediation analysis. Our results indicated that the rise in DunedinPoAm levels indirectly accounted for 9.0% of the overall impact of B2M levels on cognitive function. As previously mentioned, the rising levels of B2M linked to aging can penetrate the BBB and damage the central nervous system through multiple pathways. Moreover, the accumulation of B2M in circulation may exacerbate the aging process, resulting in further decline of cognitive function. These findings underscore the significant significance of B2M as a pro-aging factor influencing cognitive performance. In elderly mice lacking endogenous B2M expression, aging-related cognitive decline is reduced, and neurogenesis is boosted (Smith et al., 2015). Additionally, systemic inhibition of transforming growth factor (TGF)-β1, a potential regulator of B2M expression, can decrease B2M levels and boost adult neurogenesis in elderly mice (Yousef et al., 2015). Similarly, diluting the concentration of pro-aging factors such as B2M in the plasma of elderly mice with a saline-albumin solution can augment neuronal regeneration capacity and boost cognitive function (Mehdipour et al., 2020). The cumulative evidence indicates that targeting B2M in plasma may represent a promising therapeutic strategy for aging-related cognitive decline. Notably, numerous longitudinal studies have indicated that a faster pace of biological aging is significantly associated with the velocity of subsequent cognitive deterioration (Belsky et al., 2020; Vaccarino et al., 2021; Sugden et al., 2022). B2M is a pro-aging factor, enabling doctors to detect at-risk individuals by monitoring plasma B2M concentrations and initiating early therapies to maintain cognitive function.

The current study has several strengths. Our data originates from an extensive collection of clinical samples in the NHANES database, with essential cognitive-related variables, especially renal function, adjusted, enhancing our findings’ validity and robustness through subgroup analysis and interaction effect testing. Furthermore, we utilized a new generation DNA methylation epigenetic clock, the DunedinPoAm, as a mediating variable, which exhibits heightened sensitivity to alterations in pro-aging factors such as B2M levels and demonstrates a stronger connection with cognitive function and vulnerability to cognitive decline. However, the current study does have several constraints. First, owing to the NHANES’ cross-sectional design, our study can only examine the cross-sectional relationship between B2M levels and cognitive function. Second, the mediation model does not establish a causal relationship but is a hypothetical framework for assessing DunedinPoAm’s mediating role. Third, the DSST score is the sole indicator of cognitive functioning evaluated in NHANES, constraining the findings’ applicability to overall cognitive performance.

## 5 Conclusion

Our results established a clear correlation between elevated B2M levels and declined cognitive performance among U.S. older adults. Furthermore, the pace of aging, as measured by DunedinPoAm, plays a mediating role in the relationship between B2M levels and cognitive performance. This study provides critical insights into the potential of B2M as a biomarker for early detection and therapeutic intervention of aging-related cognitive decline.

## Data Availability

The datasets presented in this study can be found in online repositories. The names of the repository/repositories and accession number(s) can be found at: https://www.cdc.gov/nchs/nhanes.
